# Study on the Relationships between Intrinsic Functional Connectivity of the Default Mode Network and Transient Epileptic Activity

**DOI:** 10.3389/fneur.2014.00201

**Published:** 2014-10-10

**Authors:** Renaud Lopes, Friederike Moeller, Pierre Besson, François Ogez, William Szurhaj, Xavier Leclerc, Michael Siniatchkin, Mathilde Chipaux, Philippe Derambure, Louise Tyvaert

**Affiliations:** ^1^UMR 1046, University of Lille 2, Lille, France; ^2^In vivo Imaging Core Facility, IMPRT-IFR114, Lille University Medical Center, Lille, France; ^3^Department of Neuropaediatrics, Christian-Albrechts-University, Kiel, Germany; ^4^Department of Clinical Neurophysiology, Lille University Medical Center, Lille, France; ^5^Department of Pediatric Neurosurgery, Fondation Ophtalmologique A. de Rothschild, Paris, France

**Keywords:** default mode network, functional connectivity, dynamic, epileptic interictal event, temporal lobe epilepsy, idiopathic generalized epilepsy, posterior cingulate gyrus, precuneus

## Abstract

**Rationale:** Simultaneous recording of electroencephalogram and functional MRI (EEG–fMRI) is a powerful tool for localizing epileptic networks via the detection of hemodynamic changes correlated with interictal epileptic discharges (IEDs). fMRI can be used to study the long-lasting effect of epileptic activity by assessing stationary functional connectivity during the resting-state period [especially, the connectivity of the default mode network (DMN)]. Temporal lobe epilepsy (TLE) and idiopathic generalized epilepsy (IGE) are associated with low responsiveness and disruption of DMN activity. A dynamic functional connectivity approach might enable us to determine the effect of IEDs on DMN connectivity and to better understand the correlation between DMN connectivity changes and altered consciousness.

**Method:** We studied dynamic changes in DMN intrinsic connectivity and their relation to IEDs. Six IGE patients (with generalized spike and slow-waves) and 6 TLE patients (with unilateral left temporal spikes) were included. Functional connectivity before, during, and after IEDs was estimated using a sliding window approach and compared with the baseline period.

**Results:** No dependence on window size was observed. The baseline DMN connectivity was decreased in the left hemisphere (ipsilateral to the epileptic focus) in TLEs and was less strong but remained bilateral in IGEs. We observed an overall increase in DMN intrinsic connectivity prior to the onset of IEDs in both IGEs and TLEs. After IEDs in TLEs, we found that DMN connectivity increased before it returned to baseline values. Most of the DMN regions with increased connectivity before and after IEDs were lateralized to the left hemisphere in TLE (i.e., ipsilateral to the epileptic focus).

**Conclusion:** Results suggest that DMN connectivity may facilitate IED generation and may be affected at the time of the IED. However, these results need to be confirmed in a larger independent cohort.

## Introduction

Epilepsy is a common neurological disease defined by the occurrence of electrically and clinically measurable epileptic seizures. Cognitive and behavioral functions may be altered, leading to severe social and professional handicap. By definition, the patient’s clinical state is altered at the time of the ictal event and during the immediate postictal period. However, functional brain impairments can also be observed during the interictal state. These cognitive impairments may be due to factors such as structural lesions, medication effects, the underlying cause of epilepsy, and/or the occurrence of interictal epileptic discharges (IEDs) observed in electroencephalography (EEG). The repetition of IEDs may be responsible for long-lasting effects on the brain’s functional plasticity and may thus lead to cognitive disturbances (1). However, a transient cognitive impairment has also been observed at the time of each IED (2–5). This effect has been noticed for both generalized spike and wave bursts and focal epileptic events.

Simultaneous recording of the EEG and functional magnetic resonance imaging (fMRI) has provided new insights into the effect of IEDs on brain function. Event-related analyses have shown that at the time of the IED, the blood-oxygen-level-dependent (BOLD) signal increases at the epileptic focus (corresponding to activation). Furthermore, the BOLD signal may decrease to a variable extent at some distance from the epileptic focus (i.e., deactivation). In group analyses, this deactivation appears to involve a specific brain functional network – the default mode network (DMN) (6–9). The DMN involves several cortical areas (such as the posterior cingulate, the precuneus, the bilateral inferior parietal lobule, and the mesial prefrontal cortex) (10–13). This network is usually activated during wakefulness at rest and deactivated during sleep or during a task that requires great attention (14). The alteration in DMN activity observed at the time of an IED suggests that epileptic activity has a direct effect on awareness and cognitive performance. Deactivation within the DMN has been observed in patients with idiopathic generalized epilepsy (IGE) (6, 15, 16) and patients with temporal lobe epilepsy (TLE) (7, 8). However, the exact pattern of DMN deactivation has not been studied with respect to the side of the focus or the exact type of IED (17).

The long-lasting effect of epileptic activity on the brain’s functional organization during a prolonged resting-state period (classically 10 min) has been investigated in network functional connectivity studies. Based on the analysis of coherent low-frequency BOLD fluctuations, several local spatial patterns [defined as resting-state networks (RSNs)] have been identified and thus constitute a new functional map of the brain (12). The DMN is one of these distinct RSNs; in healthy subjects, its intrinsic connectivity is characterized by low intra- and inter-individual variability (18). Moreover, structural imaging analysis suggests that RSNs (such as the DMN) reflect anatomic connectivity (19, 20). DMN connectivity is low in IGE patients, relative to healthy controls (21–24). Similar patterns of low DMN connectivity have been observed in TLE patients (25–28). Pittau et al. (29) also demonstrated that the mesial structures in TLE patients were less connected to the DMN.

Studies of DMN connectivity and epilepsy have been performed on 10-min resting-state blocks (based on the assumption that DMN connectivity is stable over 10 min). However, there is increasing evidence to suggest that the intrinsic connectivity and spatial extent of the RSNs fluctuates in a periodic manner (30–35). Furthermore, epileptic activity may affect these fluctuations (36). Characterization of these influences is crucial for better understanding the epileptic discharge and its relationship with cognitive disturbances in patients with epilepsy.

In the present study, we assessed the dynamic changes in DMN intrinsic connectivity and its relation to epileptic activity. We hypothesized that DMN connectivity is affected by the occurrence of IEDs (i.e., connectivity differs before, during, and after IEDs). However, these changes may depend on the type of IED. To better characterize these effects, we chose to evaluate two different types of IEDs: (i) generalized spike and slow-wave bursts and (ii) left focal temporal epileptic spikes. A generalized spike and wave burst might affect the connectivity of the DMN on both sides of the brain, whereas, focal IED might lead to more lateralized changes in the DMN. Our objective was to better understand the relationship between the occurrence of IEDs and the disturbance of RSNs.

## Materials and Methods

### Subjects

We retrospectively selected patients registered in the EEG–fMRI databases in the Department of Clinical Neurophysiology at Lille University Medical Center (Lille, France) (from April 2011 to December 2013) and the Department of Neuropaediatrics at the University Hospital of Kiel (Kiel, Germany) (from May 2006 to December 2009).

The inclusion criteria were related to the number and type of IEDs. We first selected IGE patients with isolated or short generalized spike and wave events recorded during the EEG–fMRI session. Secondly, we selected focal epilepsy patients with unilateral temporal IEDs. Patients were suffering from epilepsy with temporal lobe involvement (TLE). In order to perform a group analysis of our data, we chose patients with IEDs from the temporal lobe on the same side of the brain (the left). Patients had to present no more than two types of IED, and only the predominant IED-related to the epileptic focus was studied. Absence or focal seizures were not considered and patients with seizures during the fMRI session were excluded from the study. Furthermore, patients selected for this study had to present at least 10 separate IEDs during the EEG–fMRI session. And, due to our constraints of the analysis explained in Section “Dynamical Functional Connectivity,” an interval of at least 80 s between each IED was required for completion of the analysis. All patients were right-handed. Data from EEG–fMRI sessions with high-amplitude movement artifacts ( >1 mm in each direction) were excluded from the analysis. Hence, patients were included in one of two groups: (i) IGE patients with typical, generalized slow-wave spikes, or short bursts on the EEG, and (ii) TLE patients with unilateral (left) temporal spikes.

All patients were receiving antiepileptic medication at the time of the study (Table 1). The patients (and their parents in minors) gave their written informed consent to participation. The study was performed according to the Declaration of Helsinski and the protocol was approved by the institutional research ethics boards.

**Table 1:** **Clinical and EEG characteristics of the patients in the IGE and TLE groups**.

Pt	Group center	Sex	Age (years)	Disease duration (years)	AEDs	Epilepsy type	Struct. MRI	IEDs			
								Nb	Mean duration (s)	Side	Type and location
1	IGE Lille	M	13	9	LMG, VPA	Childhood absence epilepsy	N	20	1.8	–	GSW
2	IGE Lille	F	23	18	TPM, BZD	Juvenile myoclonic epilepsy	N	14	2.5	–	GSW
3	IGE Kiel	F	13	10	VPA, LTG, LEV	Myoclonic absence epilepsy	N	12	<1	–	GSW
4	IGE Kiel	M	6	1	VPA, TPM, LEV	Myoclonic absence epilepsy	N	12	<1	–	GSW
5	IGE Kiel	M	10	6	LTG, ESM	Childhood absence epilepsy	N	16	<1	–	GSW
6	IGE Kiel	M	5	1	LTG, ESM	Childhood absence epilepsy	N	17	2.5	–	GSW
7	TLE Lille	F	14	10	OXCBZ, TPM, VPA	Temporo-occipital	Temporo-occipital DNET	14	<1	Left	Temporo-occipital spikes
8	TLE Lille	F	20	17	LCM, BZD	Fronto-temporal	N	22	<1	Left	Fronto-temporal spikes
9	TLE Lille	F	45	15	OXCBZ, TPM, BZD	Temporo-perisylvian	N	14	<1	Left	Temporo-perisylvian rhythmic theta bursts
10	TLE Lille	F	33	14	LMG, TPM, LCM	Temporo-perisylvian	Temporo-insular dysplasia	14	<1	Left	Temporo-perisylvian spike and waves
11	TLE Lille	F	22	9	CBZ, LCM, ZNS, BZD	Temporo-occipital	N	12	2.4	left	Temporo-occipital polyspikes
12	TLE Lille	F	18	9	LMG, LVT, BZD	Temporal	N	10	<1	Left	Temporal spikes

Mean ± SD		18.5 ± 11.4	9.9 ± 5.5							

*Pt, patient; AEDs, antiepileptic drugs; OXCBZ, oxcarbazepine; TPM, topiramate; LMG, lamotrigine; VPA, valproate; LCM, lacosamide; LVT, levetiracetam; ZNS, zonisamide; BZD, benzodiazepin; ETSM, ethosuximide; IEDs, interictal epiletiform discharges; N, normal; GSW, generalized spike and waves; Nb, number; M, male; F, female; DNET, dysembryoplastic neuroepithelial tumor; Struc., structural*.

### EEG–fMRI acquisition

In Lille, the EEG signal was recorded using 25 separate scalp MRI-compatible electrodes placed according to the international 10–20 system. In Kiel, a 30-electrodes Easycap system (Falk-Minow Services, Herrsching-Breitbrunn, Germany) was used. In both cases, FCz was the reference. To improve patient comfort and reduce movement artifacts, the head was maintained in position with foam cushions. Data were transmitted via an optic fiber cable from a Micromed amplifier (Micromed, Italy, 5 kHz sampling rate) in Lille and from a BrainAmp-MR amplifier (Brain Products Co., Munich, Germany, 5 kHz sampling rate) in Kiel to the EEG monitor located outside the scanner room. The EEG was recorded with a 1024 Hz sampling rate.

In both centers, functional imaging was performed with a 3 T MRI scanner (Achieva Philips, Best, The Netherlands) and a standard, 8-channel SENSE head coil. A T1-weighted structural image (voxel dimensions: 0.8 mm × 0.8 mm × 1.3 mm; slices: 130; matrix: 288 × 288; TE: 2 ms; TR: 20 ms; flip angle: 30°) was used for superposition on the functional images. For functional data, six or seven 10-min runs were acquired with a T2*-weighted EPI sequence (voxel dimensions: 4 mm × 4 mm × 4 mm; slices: 34; matrix: 64 × 64; TE: 35 ms; TR: 2000 ms; flip angle: 90°; volumes: 300) in Lille and with a 15-min T2*-weighted EPI sequence (voxel dimensions: 3.125 mm × 3.125 mm × 3.79 mm; slices: 30; matrix: 64 × 64; TE: 45 ms; TR: 2250 ms; flip angle: 90°; volumes: 540) in Kiel.

The patients were recorded at rest for up to 2 h inside the MRI. They were instructed to rest and to keep their eyes closed throughout the whole MRI session. The EEG signal was used by a neurologist to monitor the patient during the entire recording. In order to display a clear EEG, the gradient artifact was corrected online using an adaptive filtering algorithm (Brain Products, Munich, Germany). Only the raw EEG was recorded. No specific drug dose step-down, sleep deprivation, or seizure induction methods were used. Patients received chloral hydrate before the MRI session.

### EEG analysis

The EEG signal was processed off-line using Brain Vision Analyzer software (Brain Products, Munich, Germany), with correction of the gradient artifact and filtering of the EEG signal (37). A 50 Hz low-pass filter was applied to remove any remaining artifacts. Independent component analysis (ICA) was used to extract the ballistocardiogram artifact (38). After correction of the EEG, a neurologist reviewed the signal. Epileptiform events were marked according to their type, location, and duration. The predominant IED type was selected according to its frequency of occurrence and to its relevance to the suspected epileptic focus.

### fMRI preprocessing

Structural data were preprocessed by Freesurfer software (v.5.1)^1^. Each subject’s structural data underwent non-uniformity and intensity correction, skull stripping, and automatic tissue classification. Preprocessing and analysis of fMRI data were performed using a combination of Statistical Parametric Mapping software (SPM12^2^; Wellcome Department of Cognitive Neurology, University College London, UK), and in-house software implemented in MATLAB v7.11 (Mathworks Inc., Natick, MA, USA). Functional image preprocessing included the removal of the first three image volumes (to avoid T1 equilibration effects), realignment, slice-timing correction (using the middle slice as the reference frame), and registration against the structural data. Nuisance signals were removed using a two-step linear regression. The first regression removed linear/quadratic trends (to account for scanner drift) and six motion parameters. The second regression removed five “nuisance signals” obtained by means of a principal component analysis of white matter and ventricle signals using the component-based noise correction (CompCor) approach (39, 40). Residual data were corrected for high temporal frequencies (low-pass filtering with a 0.1 Hz cut-off).

Lastly, structural and functional preprocessed data were spatially normalized to match the Montreal Neurological Institute (MNI) template. For that, a non-linear registration was applied to match the preprocessed T1-weighted data to the MNI template, using SPM software. Then, the transformation was applied to preprocessed fMRI data and resampled by spline interpolation into a final voxel size of 3 mm × 3 mm × 3 mm.

### The DMN mask

Connectivity-based methods have been used to detect functionally connected brain networks with high consistency and reproducibility across subjects and sessions (41). Graph theory has been recently used to study the topological organization of these brain networks, by modeling the brain as a collection of nodes (e.g., brain regions) and edges (e.g., connectivity) [see Ref. (42), for an excellent review]. In this study, we are interested in the topological organization of DMN using a graph theory-based approach.

To study DMN intrinsic connectivity, we had first to identify the network. In view of the small number of study participants and the “double dipping” issue (43), the DMN was identified using data from healthy volunteers (part of the 1000 Functional Connectome Project, a publicly available collection of resting-state fMRI datasets from a number of laboratories around the world).^3^ The corresponding institutional review boards have approved or provided waivers for the submission of anonymized data, which were obtained after provision of written, informed consent by each participant. We selected 198 healthy volunteers (76 males and 122 females, aged 18–26) from the Beijing dataset. The DMN was then identified using group-level spatial ICA, as implemented in the GIFT toolbox.^4^ We used a low-order model (20 components) to extract the DMN into one component using the infomax ICA algorithm repeated 10 times. After thresholding to *z*-score >2, seven clusters (or regions of interest) per hemisphere were identified by selecting 30 voxels around the peak of each cluster. Thus seven regions per hemisphere were defined in MNI space: the precuneus/posterior cingulate cortex (PCC) (MNI coordinate: 8, −53, 15 and −8, −55, 18), inferior parietal lobule (IPL) (MNI coordinate: 48, −64, 31 and −45, −69, 32), middle temporal gyrus (MTG) (MNI coordinate: 56, −2, −25 and −57, −6, −22), parahippocampal gyrus (PHG) (MNI coordinate: 27, −21, −23 and −26, −26, −20), temporal pole (TP) (MNI coordinate: 40, 21, −37 and −41, 19, -37), middle frontal gyrus (MFG) (MNI coordinate: 6, 54, −9 and −7, 51, −11) and dorsal medial prefrontal cortex (dMPFC) (MNI coordinate: 20, 39, 46 and −21, 32, 47).

### Dynamical functional connectivity

Functional connectivity is commonly computed by estimating the covariance between regions. However, estimation of the covariance matrix can be a difficult statistical problem for two reasons: (i) the positive definite constraint on the matrix and (ii) the fact that there are more connections than samples. Connectivity can also be measured by estimating the inverse covariance matrix (the precision matrix) between regions under sparsity constraints. The zero entries in this matrix correspond to conditional independence between regions if the data are normally distributed. This procedure amounts to limit the number of edges in graphical models. The graphical least absolute shrinkage and selection operator (GLASSO) (44) is an extension of the least absolute shrinkage and selection operator (45) for estimating a sparse precision matrix with l1-constraint. This approach was recently applied to estimate brain region functional connectivity in a small number of samples (46–48).

**Figure 1:**
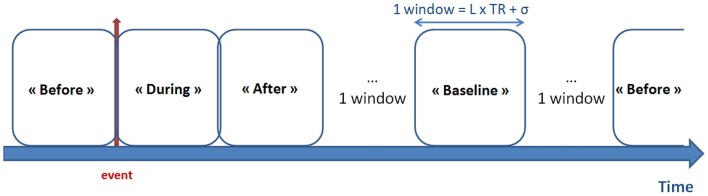
**Definition of the WOIs**. The red arrow represents the timing of the epileptic event (according to EEG data). Four types of window were defined: “before,” “during,” and “after” the epileptic event, together with the “baseline” period (i.e., with no epileptic events LTR seconds before and after the window). Tapered windows were used by convolving a rectangle (length: L TRs) with a Gaussian (σ = 2 TRs).

The mean BOLD time courses were extracted from the *N* = 14 defined DMN regions. The functional connectivity was estimated for four conditions, corresponding to windowed segments of the time courses [referred to as “before,” “during,” “after,” and “baseline” windows of interest (WOIs)]. Figure 1 shows the selection of a WOI of length L. We used a tapered window created by convolving a rectangle (length: L TRs) with a Gaussian (σ = 2 TRs). Assuming that an event occurred at time *t*, the “before” WOI corresponded to a window from *t*–L TRs to *t* TRs, with a “during” WOI from *t* TRs to *t* + L TRs, an “after” WOI from *t* + 2L TRs to *t* + 3L TRs, and a “baseline” WOI from *t* + 4L TRs to *t* + 5L TRs. No other events occurred during these periods or after 2L TRs from the end of “baseline” period. In the experiments, we varied the length L from 6 to 10. In view of our constraints, use of broader WOIs generated too few events.

According to the literature (49–51), early BOLD response can precede the IEDs observed on scalp EEG. Supplementary analyses were conducted to check this effect. We performed additional EEG–fMRI analyses using different timings of hemodynamic response function (HRF) preceding the IEDs (−9s, −7s, −5s, −3s, and 0) [for method see Ref. (49, 50)]. None of our subjects had significant BOLD changes according to these early convolved HRFs.

Next, for each condition, the functional connectivities between each region were computed using the windowed time series from corresponding WOI of length L. The covariance matrix was estimated from the regularized precision matrix using the GLASSO approach (i.e., the Ω that maximizes the penalized Gaussian log-likelihood) by using a coordinate descent optimization procedure (44):

(1) $$\log\;\det\left(\Omega \right)-\mathrm{tr}\left(\sum \Omega \right)-\lambda {∥\Omega ∥}_{1}$$

where “det” means the determinant of Ω and λ is the regularization parameter optimized for each subject after evaluating the log-likelihood of unseen data (windowed covariance matrices from the same subject) in a cross-validation framework. For each condition, the regularized covariance matrix *W* was Fisher-transformed to improve normality.

For each subject, we applied a general linear model on the covariance matrices estimated for each condition in the previous step, in order to investigate its contrast effect size. Thus, four “mean regularized covariance matrices” *M* (corresponding to contrast effect results from each condition) were obtained for each subject. These matrices will be subjected to later inter-condition analyses. This step was repeated for different lengths of window [i.e., Ref. (6, 8, 10)].

### Statistical analysis of static connectivity

Although the direct comparison of the static connectivity between TLE and IGE groups was not the main aim of this study, we performed a common graph theory-based approach using static functional connectivity. We wished to see if such an analysis of our patient groups would be consistent with previous results in the literature.

We compared DMN intrinsic functional connectivity in the TLE and IGE groups by considering whole time series. For each subject, the average whole time series of each DMN regions (see The DMN Mask) was computed and we measured the degree of DMN integration, as described in Marrelec et al. (52). A Bayesian numerical sampling scheme was used for the inference of integration measures in a group analysis. The integration was approximated from 1000 samples.

### Statistical analysis of dynamic connectivity

In this section, we were interested in DMN intrinsic connectivity for TLE and IGE groups, separately. We compared the four conditions for each group of patients. The statistical comparison of connectivity between conditions was performed using graph theory. The mean covariance matrices *M* were represented as a graph *G* = (*V*, *E*), where *V* = {*V_i_*}*_i_*_=1, … ,_ *_N_* are the nodes (brain regions) and *E* = {*E_i_*}*_i_*_=1, … ,_ *_N_*_;_ *_j_*_=1, … ,_ *_N_* are the elements of the matrix corresponding to edge weights (or connections). We used a multiscale approach to investigate the differences between the four conditions (“before” vs. “during” vs. “after” vs. “baseline” WOIs) at the network, node, and edge levels.

At the network level, the DMN integration *I* for each condition and a given subject *s* was computed to capture the overall level of statistical dependence within the network:

(2) $$I_{i,s}=\frac{1}{2}\log\left(\det\left(M_{i,s}\right)\right),\;i=1,\;\ldots ,\;4$$

where *M_i,s_* is the covariance matrix estimated for condition *i* and subject *s*, and “det” means the determinant of the matrix.

A non-parametric paired difference test (Wilcoxon’s signed-rank test) was applied on integration measures. The resulting *p*-values were corrected at *p* < 0.05 using false discovery rate (FDR), yielding increased and decreased integrations between two conditions.

At the node-level, two topology measures were used: the node strength (i.e., the sum of weights of links connected to a node *i*, also known as weighted node degree):

(3) $$k_{i}=\sum_{j=1}^{N}E_{i,j}$$

and the clustering coefficient of node *i*, which is the average “intensity” of triangles around a node and reflects the prevalence of clustered connectivity around a node:

(4) Ci=∑j,h=1N(wijwihwjh)13ki(ki−1)

The clustering coefficient was computed according to the Brain Connectivity toolbox.^5^

A Wilcoxon signed-rank test was applied to these measurements and the resulting *p*-values were FDR-corrected at *p* < 0.05 (indicating differences in inter-node connectivity values when comparing two conditions).

At the edge level, we focused on comparing DMN intrinsic connectivity at a pairwise level. A network-based statistics (NBS) approach (53) was used to identify pairs of regions between which the strength of connectivity was altered in the conditions of one group. NBS approach has already been used to show altered structural connectivity in absence epilepsy (54) and TLE (55). For the comparison of two conditions for *S* subjects, a primary threshold (*p* = 0.05) was first applied to a *t*-statistic computed from the matrices {*M_s_*}*_s_*_=1 …_ *_S_*. This *t*-statistic was computed for each graph edge, in order to determine any connected components and their size. A family wise error-corrected *p*-value was then assigned to each network using permutation testing. The condition label on each covariance matrix was permuted (10,000 permutations) constrained for repeated (within-subject) measures to assess statistical significance between two conditions. Lastly, only statistically significant networks (with a *p*-value of 0.05 corrected for multiple comparisons) were selected.

The brain networks were visualized with BrainNet Viewer software^6^ (56).

## Results

### Subjects

In view of the restrictive inclusion criteria, only 12 patients were eligible for inclusion (6 IGE patients and 6 TLE patients). Details on all subjects included in the study are listed in Table 1. Indeed, in Lille and Kiel databases patients’ recruitment was based on IEDs frequency. Then only active epileptic patients were recorded in order to maximize the chance to observe IEDs during the MRI session. In our study, to perform our analysis an interval of at least 80 s between each IED was required. This criterion was rarely obtained. In the IGE group, we included four males and two females (mean ± SD age: 11.5 ± 6.5 years; mean epilepsy duration: 7.5 ± 6.4 years). Based on the International League against Epilepsy criteria (57), three of them were suffering from childhood absence seizure, two were suffering from myoclonic absence epilepsy, and one was suffering from juvenile myoclonic epilepsy (Table 1). The structural MRI datasets were normal in all patients. The TLE group comprised six females (mean ± SD age: 25.3 ± 11 years; mean epilepsy duration: 12.3 ± 3.4 years). All six were suffering from epilepsy involving the left temporal lobe with left temporal IEDs. The structural MRI datasets were normal in four cases (patients 8, 9, 11, and 12). Patient 10 had a left temporo-insular dysplasia and patient 7 presented a left temporo-occipital dysembryoplastic neuroepithelial tumor.

### DMN connectivity at the network level

**Figure 2:**
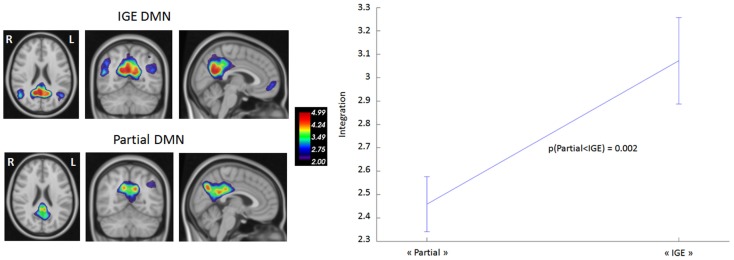
**DMN functional connectivity in TLE vs. IGE groups**. The DMN integration (dimensionless) was computed for each subject of the two groups. A Bayesian numerical sampling scheme was used for the inference of integration measures in a group analysis. The integration was approximated from 1000 samples. The error bars indicated the standard deviation of the 1000 samples. There was significantly less integration in the TLE group than in the IGE group (right panel). To illustrate this difference, DMN was estimated for each group using the same groupICA approach than the Section “DMN mask” (left panel). Maps were thresholded (*z*-score > 2).

We first compared DMN intrinsic functional connectivity in the TLE and IGE groups by considering whole time series. Figure 2 shows that DMN integration in the TLE group was significantly lower than in the IGE group. The error bars indicated the standard deviation of the 1000 samples. This result was confirmed by inspection of the DMN maps for the two groups (thresholded for a *z*-score >2) using the GroupICA approach (the same method as used for healthy volunteers in Section “The DMN Mask”) (Figure 2). In the TLE group, the DMN was composed of the PCC and the right IPL. In the IGE group, it was composed of the PCC and the bilateral IPL, MTG, PHG, and dMPFC regions. Furthermore, we observed negative connections between the DMN and the left superior frontal and inferior frontal gyri for the TLE group. DMN anti-correlated network was larger in the IGE group (with the occipital pole and the bilateral inferior frontal and superior insula gyri) than in the TLE group.

We then studied DMN intrinsic functional connectivity in the four WOIs. Functional connectivity was estimated using a window size of eight TRs (from 16s to 18s). When varying the window size from 6 to 10 TRs, we did not find any significant differences between the respective results. Hence, a window size of eight TRs was chosen because it provided a good trade-off between the ability to resolve dynamic changes and the ease of computing regularized covariance matrices. Table 2 shows the inter-condition differences in DMN integration. In the TLE group, significantly stronger integrations were found in the “before” and “after” WOIs than in the “during” and “baseline” WOIs. There were no significant differences between “during” and “baseline” WOIs. These data indicate that the most significant changes occur before and after an epileptic event. However, the DMN intrinsic connectivity was similar during an IED and during the “baseline” period. In the IGE group, we did not observe any significant inter-conditions difference in DMN integration. Although we found greater DMN integration in the “before,” “during,” and “after” WOIs than in the “baseline” WOI, the differences were not significant (with *p*-values of 0.08, 0.16, and 0.18, respectively).

**Table 2:** **Mean integration of the DMN network for the TLE and IGE groups under different conditions and for a window size of eight TR**.

	Before events	During events	After events	Baseline
TLE patients	1.09 ± 0.14	0.89 ± 0.13^(+)(x)^	1.06 ± 0.09	0.90 ± 0.10^(+)(x)^
IGE patients	1.15 ± 0.18	1.08 ± 0.20	1.09 ± 0.23	1.01 ± 0.20

*(+) and (x) indicate significantly different (*p* < 0.05) means in a paired Wilcoxon signed-rank test when comparing the designated column with the “before” and “after” columns, respectively*.

### DMN connectivity at the node level

**Figure 3:**
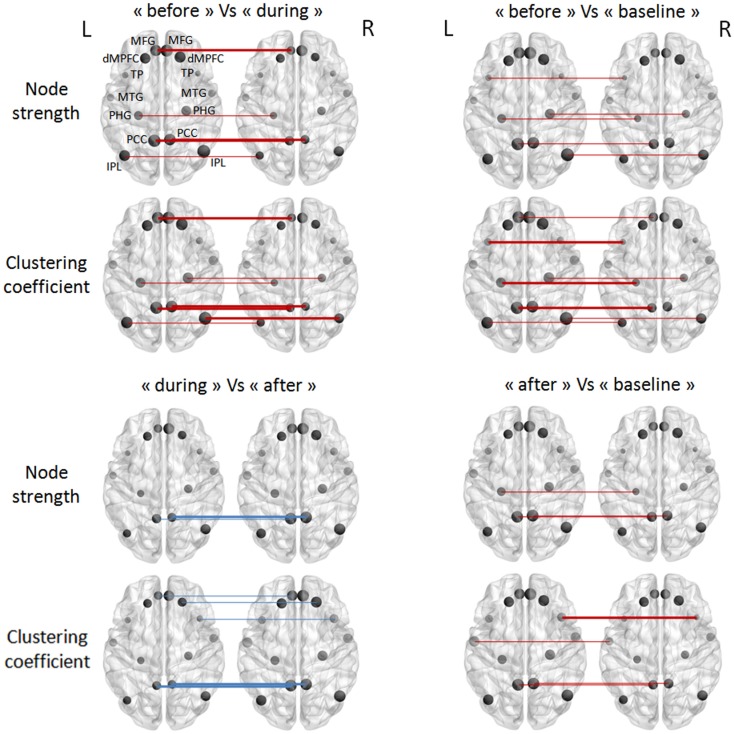
**Pairwise comparisons of node strength and the clustering coefficient in the TLE group (in a Wilcoxon signed-rank test)**. The node sizes correspond to the mean network measure being tested. Colors indicated the direction of change (the red lines mean a decrease, and the blue lines an increase, from the first condition to the second). Different line style indicated significance (thin for FDR-corrected *p*-values <0.1 and thick for FDR-corrected *p*-values <0.05).

After studying connectivity at the network level, we sought to determine which nodes (or brain regions) were most involved in the inter-condition differences described above. Two topological measures were used to quantify the nodes’ function: node strength and the clustering coefficient. Figure 3 summarizes the results obtained for the TLE group; no significant differences between “during” and “baseline” WOIs were observed for either measure. The “before” and “during” WOIs differed most in terms of numbers of nodes (4), and differed significantly in terms of node strength and the clustering coefficient. According to both measures, the PCC and left MFG regions had greater connectivity before an IED than during an IED. Likewise, the right IPL region had greater connectivity with its neighborhood. Intrinsic hyperconnectivity of the DMN was also observed in the “before” WOI comparing with the “baseline” WOI but this phenomenon involved different brain regions. Only regions from the left hemisphere (PCC–PHG–TP) differed in terms of clustering coefficient. Lastly, hyperconnectivity was also observed for regions when comparing the “after” condition on one hand with the “during” and “baseline” conditions on the other. However, the hyperconnectivity in the “after” condition was less intense than for “before” condition. We observed that the left and right PCC regions were hubs of hyperconnectivity after (but not before) the IED.

**Figure 4:**
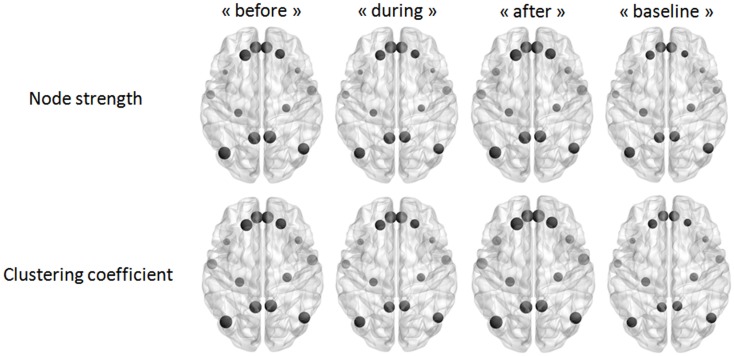
**Pairwise comparisons of node strength and the clustering coefficient in the IGE group (in a Wilcoxon signed-rank test) thresholded at FDR-corrected *p*-values <0.05**. The node sizes correspond to the mean network measure being tested.

In the IGE group, there were no significant inter-condition differences in node-level intrinsic connectivity of the DMN (Figure 4).

### DMN connectivity at the edge level

**Figure 5:**
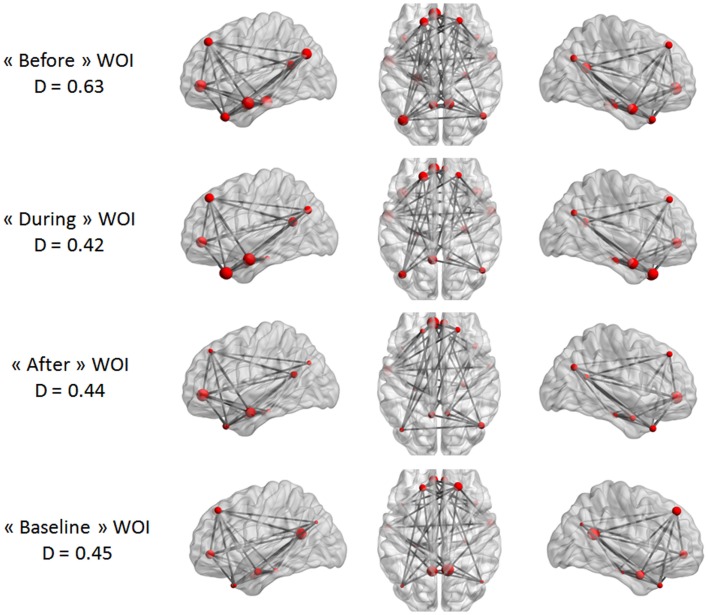
**Maps of DMN intrinsic connectivity in the TLE group**. The node sizes correspond to the number of connections for a node and the gray lines show significant connections between pairs of regions (FDR-corrected *p* < 0.05). The variable *D* represents the network density (i.e., the number of significant connections divided by the total number of connections).

**Figure 6:**
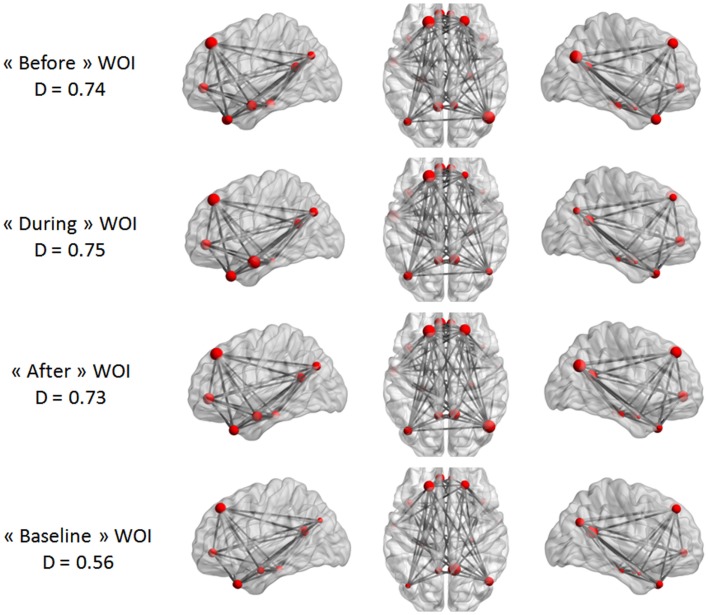
**Maps of DMN intrinsic connectivity in the IGE group**. The node sizes correspond to the number of connections for a node and the gray lines show significant connections between pairs of regions (FDR-corrected *p* < 0.05). The variable *D* represents the network density (i.e., the number of significant connections divided by the total number of connections).

Our last analysis focused on DMN intrinsic connectivity at the edge (connection) level. In a first step, a one-sample *t*-test was performed on each condition using NBS. For one condition, we compared the mean regularized covariance matrices from all subjects. Figures 5 and 6 show pairs of regions that were significantly connected (*p* < 0.05, corrected) during each condition in both epilepsy groups. For TLE and IGE groups, the DMN intrinsic connectivity was relatively high for all conditions. We did not see a condition with very few connected regions. For TLE group, it seemed that the temporal regions (TP–MTG and PHG) showed greater connectivity in the “before” and “during” WOIs than in the “after” and “baseline” WOIs. For IGE group, it seemed that the DMN intrinsic connectivity was higher in the “before,” “during,” and “after” WOIs than in the “baseline” WOI.

**Figure 7:**
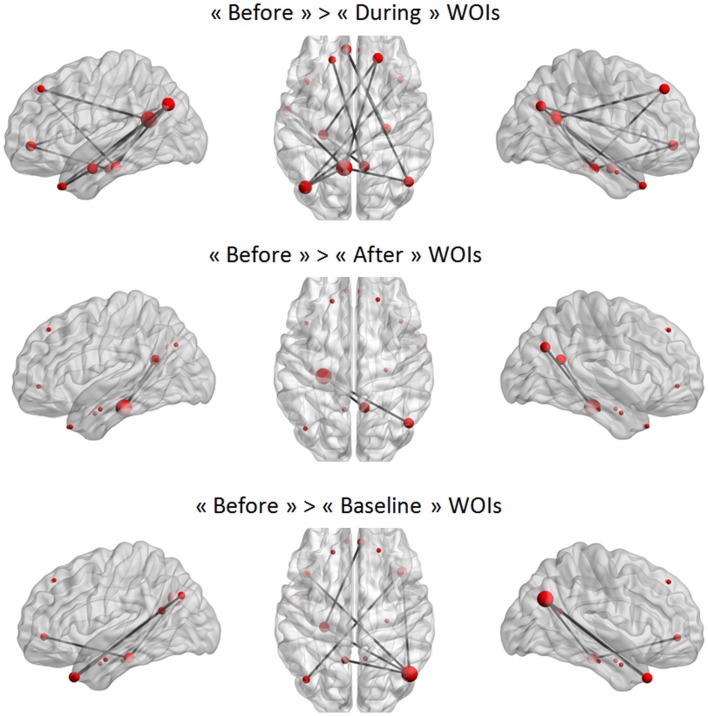
**Significant differences between two WOIs in the TLE group**. The node sizes correspond to the number of connections for a node and the gray lines show significant connections between pairs of regions (FDR-corrected *p* < 0.05).

In a second step, the conditions were compared in a paired *t*-test to statistically validate these observations (again using NBS). The only statistically significant difference was that between the “before” WOI and all other WOIs in the TLE group (Figure 7). A larger difference between “before” and “during” WOIs than “after” or “baseline” WOIs was observed (especially for the left PCC node). The PCC was more strongly connected with the parietal, frontal, and temporal regions in the “before” WOI than in the other WOIs. Although differences were observed in the two hemispheres, the number of significant connections was higher in the left hemisphere than in the right hemisphere. This difference was especially marked for connections between extra-temporal and temporal regions. Only connections between the left PHG and the right PCC and right IPL were significantly stronger in the “before” WOI than in the “after” WOI. Lastly, the “before” WOI had greater connectivity between temporal and extra-temporal regions than the “baseline” WOI. Indeed, differences were observed for connections involving the left and right TPs and the left PHG. The “after” WOI did not differ significantly from the “during” and “baseline” WOIs at this level of connectivity.

## Discussion

The present study focused on two different types of epilepsy (TLE and IGE) with a common clinical feature: reduced responsiveness at the time of the epileptic discharge. In both types of epilepsy, the DMN is known to be affected by IEDs (5–7, 13–15). We first compared DMN connectivity (based on a standard analysis) in TLE and IGE patients. The decrease in DMN functional connectivity was lateralized (on the focus-side) in TLE patients and was diffuse in IGE patients. By performing a time-resolved analysis of changes in DMN intrinsic connectivity, we found that the overall level of connectivity increased before the onset of the IED in both IGE and TLE patients. This effect was more marked in the TLE group with recovery of a spatially bilateral DMN connectivity pattern prior to the IED. In TLE patients, the DMN connectivity increased after the IED (predominantly in the PCC) and then returned to baseline values (baseline DMN configuration).

### Baseline DMN connectivity and epilepsy

Before studying the time course of change in the DMN connectivity, we wished to confirm the literature reports of alterations in DMN connectivity in IGE and TLE patients by analyzing the resting-state (i.e., the period free of IEDs). We also sought to determine whether the DMN connectivity pattern differed as a function of the type of epilepsy. Firstly, we observed that the DMN connectivity pattern remained bilateral in IGE patients. In the TLE group, the level of DMN connectivity was clearly lower than in the IGE group (Figure 2); it involved all the nodes but had a right predominance pattern: the left IPL, MTG, and PHG did not form part of the DMN. These results agree with literature data on both types of epilepsy. In TLE patients, only one study failed to observe an asymmetric pattern with lower functional connectivity on the focus-side (26). However, the authors in the latter study did not monitor the resting-state period; residual IEDs (or even seizures) may have occurred and thus affected the analysis. Zhang et al. (28) and Luo et al. (22) observed low connectivity in the dMPFC and very low or even no connectivity in the mesial temporal lobe and the superior temporal gyrus ipsilateral to the epileptic focus-side. These researchers observed this pattern in right and left TLE patients. Although the right TLE patients showed low functional connectivity in right and left mesial temporal lobes, the left TLE patients had only low functional connectivity in the left mesial temporal lobe. Greater functional connectivity in the posterior cingulate gyrus was only observed in patients with right TLE. Frings et al. (25) confirmed the presence of an asymmetric DMN disconnection after observing low functional connectivity between the precuneus and temporal structures in left TLE patients. In studies using region of interest methods, the overall DMN connectivity in IGE patients is found to be lower than in controls (22–24). Using ICA, Wang et al. (58) observed low functional connectivity in the dMPFC, precuneus and right angular gyrus, and high connectivity in the PCC. These MRI studies did not monitor the EEG. As mentioned above, the epileptic event might directly interfere with overall DMN connectivity. Recently, Kay et al. ’s (21) EEG–fMRI study focused on IED-free periods and observed low overall connectivity in the posterior part of the DMN (the PCC, the left and right inferior parietal areas and, to a lesser extent, the left and right prefrontal gyri). Our present results are similar to the literature findings, even though we had small numbers of patients in each group. In TLE and IGE patients, our present results and the literature data emphasize the effect of chronic epileptic discharges on the overall functional connectivity of the brain. During clinical epileptic events in IGE and TLE, cognitive impairment is associated with changes in the DMN configuration. Recurrent IEDs and/or seizures may account for the observed reorganization of DMN connectivity in both types of epilepsy. One can speculate that low functional connectivity in the DMN in IGE and TLE patients may result in cognitive impairment during the interictal period. Although we observed abnormally low DMN connectivity in both types of epilepsy, TLE was further characterized by a marked lower temporal connectivity in the left hemisphere (the left PHG, MTG, IPL, and PCC). This clear, focus-side decrease in DMN connectivity in TLE (relative to IGE) rules out the hypothesis whereby differences in DMN connectivity are due to differences in the mean age of IGE and TLE groups. The mechanisms underlying the changes in the DMN may vary according to the type of epilepsy. In IGE, seizures and IEDs involve widespread, bilateral subcortical, and cortical areas at the same time via thalamo-cortical and cortico-cortical networks (6, 51, 59–65), even though focal onset can sometimes be observed (66, 67). DMN connectivity could be affected by recurrent activation of these thalamo-cortical and cortico-cortical networks on both sides of the brain. TLE is defined by the emergence of an aberrant epileptic network in the temporal lobe, which involves unilateral, local anatomical, and functional reorganization. This local epileptic activity can affect distant areas of the brain (such as the DMN), suggesting that focal epileptic discharge has a widespread asymmetric effect (predominantly on the focus-side).

### IEDs and the intrinsic dynamic connectivity of the DMN

The main aim of this study was to evaluate the changes in intrinsic DMN connectivity before, during, and after the IED. Indeed, the standard method for measuring DMN connectivity is based on observing the resting-state period for around 10 min. It is assumed that brain activity and RSN connectivity are stable during this time. However, it is known that brain states cannot necessarily be controlled during the resting-state; subjects are submitted to variable external and internal stimuli that will affect their brain processes in different ways. The assumption made in many studies (that ongoing activity is sufficiently random to be averaged) induces a bias. It is clear that brain activity displays specific features that are not random over time (68). Recent research has demonstrated that RSN connectivity also changes over time, with periodic fluctuations reflecting different brain states (30–34). This time course is random. Conventionally defined RSNs correspond to the sum of the brain’s different network configurations. Over long time scales, RSNs may reflect anatomic connectivity (19, 20). Over short time scales, different configurations of each RSN can be observed but they are always based on the brain’s underlying functional structure. Based on this concept of dynamic changes in RSN connectivity, it is possible to evaluate the effect of the IED or seizure on the RSNs’ respective time courses. Liao et al. (36) were the first to explore this concept with a view to better understanding the network properties involved in the onset and offset of absence seizures. The researchers particularly studied the dynamic interactions between DMN and the thalamic network at and around the time of the epileptic event. They found a negative correlation between the two networks at the time of the absence seizure and a positive correlation during the baseline period. These results confirmed previous EEG–fMRI studies (based on event-related analysis) that reported an anti-correlated BOLD signal pattern in the DMN and in the thalami (overall DMN deactivation and activation of the thalami) at the time of the absence seizure. In our present work, we chose to evaluate the effect of the epileptic event on DMN intrinsic connectivity during different time windows (before, during, and after the IED and at baseline). Although our initial analysis showed that overall DMN connectivity is affected during IED-free periods, it did not provide information on the specific effect of the IED on connectivity.

#### DMN connectivity changes during the IED

At the time of the IED, RSN connectivity is necessarily affected by the epileptic event itself. Indeed, we hypothesized that structures displaying a significant increase or decrease in the BOLD (i.e., a hemodynamic response) at the time of the IED are highly connected. Previous event-related EEG–fMRI studies revealed a transient decrease in the BOLD signal in DMN areas at the time of generalized spike and slow-wave discharges and the focal TLE spikes (6–8, 15, 16). We assumed that the intrinsic connectivity of the DMN would be affected, with greater connectivity in the nodes in deactivated brain areas and decreased connectivity in nodes in non-deactivated areas. In fact, we did not find any difference in DMN connectivity when comparing the “during” period and the baseline period. This means that in IGE patients, the DMN connectivity remained stable and uniform including all the usual DMN nodes. This result is consistent with the findings of event-related EEG–fMRI studies, i.e., bilateral, symmetric DMN deactivation at the time of generalized spike and slow-wave bursts. In TLE, DMN connectivity was low and asymmetric during IEDs. The left temporal and parietal DMN nodes (the PHG, IPL, and MTG) were disconnected from the usual DMN components. These nodes are probably affected by the specific temporal lobe epileptic network involved in IED generation. The similar connectivity configurations in the “during” and “baseline” conditions suggest that the baseline DMN reflects functional (and probably structural) alterations caused by recurrent epileptic discharges.

#### DMN connectivity changes before the IED

In both TLE and IGE, we always observed greater DMN connectivity several seconds before the onset of the IED (in the −8 TR to 0 s WOI). In the TLE group, the DMN configuration “before” the IED was characterized by an increase in DMN connectivity in the left PCC and dMPFC (relative to the “during” configuration). When compared with the “baseline” connectivity pattern, DMN connectivity was particularly enhanced in the PCC, left MTG, left PHG, and left TP. In the IGE group, this effect was not statistically significant. However, we observed a trend toward greater DMN connectivity during the “before” window than during other time windows (*p* = 0.008). In view of the lack of statistical power, we were not able to identify specific structures that may be particularly affected by this increase in connectivity. This statistical issue may be due to the small size of the study population and the small differences between a normal DMN configuration and the IGE configuration.

Changes in DMN connectivity observed before the electrical epileptic event at the scalp is consistent with previous observations of metabolic changes before EEG events. Indeed, several studies have demonstrated that oxygenation and BOLD changes may occur several seconds before the generalized spike and slow-waves or before focal spikes (49–51, 69, 70). Early BOLD changes could be observed up to 9 s before the EEG event. This long delay suggests that mechanisms other than “invisible” electrical changes may precede scalp IED changes, i.e., primary hemodynamic and metabolic events may occur before the electrical changes. Likewise, Zhao et al. (71, 72) used optical imaging of the cortical surface to observe isolated oxygenation changes before the seizure onset. If focal IEDs and seizures can indeed be triggered or conditioned by an early hemodynamic and/or metabolic event, the latter appears to only affect a small, focal, cortical area (50, 71). More recently, a dynamic time course study (66) reported a significant BOLD signal increase in DMN structures (the orbitofrontal, cingulate gyrus, lateral parietal, and precuneus areas) more than 5 s before IEDs in absence seizures. In contrast, the thalami were only involved later on in the absence seizures. Our observation of increased connectivity in DMN agrees with these findings. However, the early change in DMN connectivity suggests the presence of a more complex process, with involvement of brain areas far from the epileptic focus. We observed increased DMN connectivity over a broad time window [−8 TRs (−18/−16 s) to 0 s], suggesting that this process may occur earlier than the early BOLD changes described in the literature (observed up to 9 s before the EEG event). Vaudano et al. has suggested that the DMN is involved in IED generation, with a causal link between early BOLD changes in DMN structures and the occurrence of IEDs (73). The latter researchers used dynamic causal modeling to investigate the involvement of the precuneus, thalamus, and prefrontal cortex in the spike and slow-wave discharges in IGE. They found that the onset of a generalized spike and slow-waves was linked to early activity in the posterior cingulate gyrus. This had already been suspected by Archer et al. (74) in five IGE patients, in whom only an negative BOLD response of the posterior cingulate gyrus was observed at the time of the IEDs. In the present study, we observed changes in the configuration of the DMN, with greater connectivity between the various DMN structures (including the posterior cingulate gyrus). This result corroborates Vaudano et al.’s finding. However, our study was not restricted to the PCC area and we further demonstrated that this early process affected other DMN nodes. Moreover, we found that this early process occurred in TLE patients and (albeit as a non-significant trend) in IGE patients. This implies that a specific configuration of the DMN is present before IEDs in both types of epilepsy. We suggest that in IGE, higher intrinsic connectivity in the DMN could activate the thalami and stimulate specific cortico-subcortical interactions required for generalized IEDs. We suggest that a similar process occurs in TLE: the change in DMN connectivity several seconds before the IED is characterized by a switch from the usual right predominance DMN pattern to a bilateral pattern (i.e., increased DMN connectivity in left TP, PHG, MTG, PCC, and dMPFC). This would mean that structures on the focus-side are transiently reconnected to other DMN nodes. This may increase the level of interaction between the DMN and the epileptic network. The IEDs in IGE and TLE may result from a particular interaction between a highly connected DMN and the epileptic network (a focalized, temporal network in TLE and a cortico-subcortical network in IGE).

This early switch in DMN configuration is not random and probably reflects a specific change in brain state. We speculate that the early DMN configuration reflects a physiologic fluctuation in brain state as a function of external or internal stimuli. This specific state may facilitate the occurrence of epileptic discharge. As mentioned above, the DMN is related to the consciousness state and is modulated by attention-demanding tasks. Interestingly, it has been firmly established that the occurrence of seizures and IEDs depends on the level of awareness (2). Although IEDs are responsible for disrupting normal function (leading to transient cognitive impairment at the time of the event) (75, 76), there is also evidence to suggest that cognitive tasks can directly affect the occurrence of IEDs (3). The level of attention also seems be related to the frequency of IEDs (2, 77, 78). Aart et al. (2) reported that the IED frequency in generalized and focal epileptic patients changed during cognitive testing. More recently, Matsumoto et al. (78) used a visual memory task to show that the IED rate fell as the gamma power preceding the IED onset increased. This high-frequency oscillation modulation was related to a memory-encoding task. Moreover, Fahoum et al. (17) demonstrated that DMN BOLD activity fell in parallel with the gamma band power in DMN components. Taken as a whole, these findings suggest that specific cognitive tasks (leading to gamma power fluctuations) or awareness fluctuations may change DMN connectivity and thus facilitate IED generation.

#### DMN connectivity changes after the IED time window

Compared with the “during IED” configuration, we observed greater connectivity of the right and left PCCs in TLE patients. There was no clear difference between the “after” and “baseline” periods. In the “during” period, we found that the PCC and the left-side temporal and parietal nodes were partly disconnected from the standard DMN nodes. Recovery of a baseline configuration means that the PCC has to be transiently hyperconnected to other DMN nodes (since the left-side temporal and parietal nodes are still disconnected). The PCC is less extensively connected to other DMN nodes during the IED, and this configuration is corrected after the IED. The role of the PCC in consciousness mechanisms has been studied; it appears to be the primary substrate for conscious awareness (79). Alteration of the PCC’s function during cingulate gyrus epilepsy or absence epilepsy is associated with loss of consciousness. Transient cognitive impairment is observed during focal and generalized IEDs (75, 76). A change in PCC function may be one explanation. Our results show that the PCC’s connectivity with other parts of the DMN may also be crucial for change in awareness during IEDs. As Laureys et al. (79) mentioned, the PCC has a pivotal role in consciousness/awareness regulation because of its anatomic position (with strong links to the anterior thalamus nucleus and the brainstem’s arousal system in the thalamus). In the present study, we failed to reproduce this effect in the IGE group. It would be interesting to investigate the involvement of the PCC in a larger number of IGE patients.

### Methodological considerations and perspectives

Our study focused on the relationship between the IED and the DMN connectivity changes. This work was based on the recent observation that RSNs fluctuates over time (30–34) and the hypothesis that the epileptic activity may affect these fluctuations. For this purpose, dynamic connectivity was estimated using a sliding window approach described and validated by Chang et al. (31). If this methodological approach was suitable for our research hypothesis, it also raised several methodological issues.

Firstly, the main limitation of the present study was the small number of patients (*n* = 6) in each group. This was explained by the severe selection criteria (at least 10 isolated IEDs with an interval of at least 80 s between each IED were required). To perform relevant statistical analysis on these small size groups, non-parametric statistical analyses were used. This study is a preliminary work and it would be useful to increase the sample size for further analyses (especially to evaluate effect of disease duration, effect of antiepileptic drugs…) and to confirm our hypotheses. Secondly, the regions of interest of the DMN were chosen using a group-level spatial ICA of 198 healthy volunteers (76 males and 122 females) aged 18–26. The IGE patients were younger than the TLE patients and the healthy volunteers. This high range of ages is related to the different types of epilepsy. Indeed, if IGE is affecting mostly infants and adolescents, TLE is affecting young adults and adults. Previous studies of difference in DMN intrinsic connectivity between children and young adults (80, 81) found similar spatial patterns, with only weaker DMN connectivity and decreased spatial extent in the dMPFC in the children. As described in Section “DMN Connectivity at the Network Level,” we found weaker DMN connectivity in the TLE group than in the IGE group. We performed the DMN integration comparisons between IGE and TLE patients using whole time series without the dMPFC node. This analysis yielded similar result. Hence, differences in mean age are unlikely to have biased our data on DMN functional connectivity changes. The wide range of the subject’s ages could also be an issue for spatial normalization of fMRI data. Visual inspection of spatial normalization results was performed to detect abrupt differences in quality of registration between children and adults and no relation was found between this spatial normalization and age of subjects. Even if the effect of brain maturation cannot be totally ruled out on our results, the difference observed between IGEs and TLEs in term of DMN functional connectivity changes is likely to be due to the type of epileptic disorders and events. The two groups also exhibit a difference in gender (six females in TLE group and two females in IGE group). Our results are unlikely to be affected by this difference as the DMN connectivity has been shown to be robust and similar between sexes (82, 83). Thirdly, to define the different windows (“baseline,” “before,” “during,” and “after”) we used the IED timing. If the “during” and “after” windows are easy to define based on the timing and the short duration of each IED, the “baseline” and the “before” window may raise more difficulties. The epileptic activity in our work is defined by the occurrence of IEDs observed on scalp EEG. Scalp EEG can be blind to deeper epileptic activities especially in TLE patients. This point is a common limitation of the literature on EEG–fMRI studies. Facing this difficulty, authors focusing on functional resting-states networks used also the period of EEG without IEDs as the baseline to avoid interferences with epileptic activity (19). In our study, we can suggest that if this deeper epileptic activity had interfered on our results, because the occurrence of this specific activity would have been random, our results would have been statistically irrelevant. We showed that especially in TLEs our results on DMN intrinsic connectivity are statistically consistent. We suggest then that deeper epileptic activity is possible but is unlikely to affect our analysis. Intracerebral EEG (iEEG) recording simultaneously with fMRI may be one way to control this “deeper” epileptic activity. However, the spatial sample of intracerebral electrodes is also limited and can provide other bias. To go further, we checked that no BOLD change can be observed before the scalp IEDs timing. Indeed previous works showed that in IGE patients and in some cases of focal epilepsy (49, 50) an early BOLD change can be observed. This early BOLD change would affect the signal during the “before” window and provide heterogeneity between patients.

The dynamic connectivity was estimated using a sliding window approach. Thus, the length (L) of the windows was an important degree of freedom. The dependency of the window of length L were investigated in the present study, but the range (*L* = {6 TRs, … , 10 TRs}) was limited due to the need of at least 4L between each IED. In fact, windows larger than *L* = 10 TRs (20 s/22.5 s) did not contain enough IEDs per subject. We varied the window size from 6 TRs to 10 TRs and did not find any statistically significant differences between the respective results (Figure S1 in Supplementary Material). We chose to report results with windows of length *L* = 8TRs because it provided a good trade-off between time resolution and the ability to compute regularized covariance matrices with a low standard deviation. Furthermore, the TR values in Kiel and in Lille were slightly different (2 s and 2.25 s, respectively). Although, a variation in TR can impact the quality of the BOLD signal, the slight overall difference (2 s for an 8 TR window) is unlikely to be significant. Moreover, we showed that larger windows (10 TRs) yielded the same results. The next step in the procedure was the estimation of DMN functional connectivity within the WOIs. Due to the relatively small window length, we decided to estimate the precision matrix (the inverse covariance matrix) rather than the covariance matrix. Use of the precision matrix is suitable when the number of connections is greater than the number of samples (as in the present study). However, different approaches could be used to study the dynamic functional connectivity of the DMN or other brain regions. In this study, the window length was a degree of freedom of our method. Other approaches to investigate the dynamic functional connectivity could be used to avoid the need of defining a window length. Previous studies were interested in dynamic functional connectivity without the need of defining WOIs. Two kinds of approaches have been used: (i) in looking for spatial patterns of dynamic connectivity and (ii) in investigating the temporal pattern of dynamic connectivity. Li et al. (84) showed that the time course of functional connectivity can be divided into quasi-stable segments via a sliding time window approach. These time segments were used to differentiate between healthy volunteers and patients with post-traumatic stress disorder. Ma et al. (85) used independent vector analysis to identify dynamic changes in spatial functional connectivity. The researchers found significantly more fluctuations and more variable patterns of spatial network concordance in schizophrenia patients than in healthy volunteers.

In the validation part of the present study, a graph analysis was used to compare the “before,” “during,” “after,” and “baseline” WOIs in both the TLE and IGE groups. A multiscale network, node and edge analysis was applied. At the network and node-levels, many different network measures can be computed by calculating measures of integration and segregation (86). We chose to limit our analysis by measuring only DMN network integration at the network level and node strength and the clustering coefficient at the node-level. We selected these criteria because they are easier to interpret. Our results revealed inter-conditions differences in DMN intrinsic connectivity in both the TLE and IGE groups.

Small window lengths were used in this study (6, 8, and 10 TRs), which causes methodological issues in the estimation of the covariance matrix. Recently developed fMRI sequences could be used to increase the time resolution (i.e., by decreasing the TR). Significant shortening of TR has been achieved using “slice” multiplexing, in which multiple slices are excited and acquired simultaneously. This approach significantly increases statistical power in functional connectivity analyses (87).

Lastly, our previous research (88) showed that a wavelet-based approach can be used to detect epileptic activity in BOLD signals alone (i.e., without the need to record the EEG). Although this method was able to detect much the same spatial patterns of epileptic activity as EEG–fMRI, only a few IEDs were detected. The dynamic changes in DMN functional connectivity could be used to improve the sensitivity and specificity of this type of approach. In the future, we intend to combine both dynamic functional connectivity and the wavelet-based approach, with a view to improve the robustness of IEDs detection in the BOLD signal.

## Conclusion

In the present study, we investigated dynamic changes in DMN intrinsic connectivity and their relation to epileptic activity. We showed that DMN connectivity is specifically affected by the IED occurrence. The greatest changes in DMN connectivity were observed before IEDs. These changes may be caused by specific brain states that facilitate IED generation. During and after IEDs, the observed change in DMN connectivity emphasized the pivotal role of the PCC in IED-related awareness fluctuations. Due to the low number of subjects, the results found in this study need to be confirmed in a larger independent cohort. Future research should investigate more precisely the relationship between the occurrence of IEDs and states of awareness/consciousness in epilepsy. The dynamic approach of the RSNs connectivity will provide a powerful tool to investigate IEDs and seizure physiopathological mechanisms.

## Conflict of Interest Statement

The authors declare that the research was conducted in the absence of any commercial or financial relationships that could be construed as a potential conflict of interest.

## Supplementary Material

The Supplementary Material for this article can be found online at http://www.frontiersin.org/Journal/10.3389/fneur.2014.00201/abstract

## Footnotes

^1^http://surfer.nmr.mgh.harvard.edu/

^2^http://www.fil.ion.ucl.ac.uk/spm/software/spm12/

^3^http://fcon_1000.projects.nitrc.org/

^4^http://mialab.mrn.org/software/gift/index.html

^5^http://sites.google.com/site/bctnet/

^6^http://www.nitrc.org/projects/bnv/

## Abbreviations

BOLD, blood oxygen level dependent; DMN, default mode network; dMPFC, dorsal medial prefrontal cortex; EEG, electroencephalogram; fMRI, functional magnetic resonance imaging; HRF, hemodynamic response function; ICA, independent component analysis; IED, interictal epileptic discharge; IGE, idiopathic generalized epilepsy; IPL, inferior parietal lobule; MFG, middle frontal gyrus; MTG, middle temporal gyrus; NBS, network-based statistics; PCC, precuneus/posterior cingulate cortex; PHG, parahippocampal gyrus; RSN, resting-state network; TLE, temporal lobe epilepsy; TP, temporal pole; WOI, window of interest.
