# Supplementary material for: Study on the Relationships between Intrinsic Functional Connectivity of the Default Mode Network and Transient Epileptic Activity
Source: Front Neurol. 2014 Oct 10;5:201. doi: 10.3389/fneur.2014.00201 (PMC4193009; doi:10.3389/fneur.2014.00201)
Supplement: Supplementary file 1 [file Presentation1.PDF]

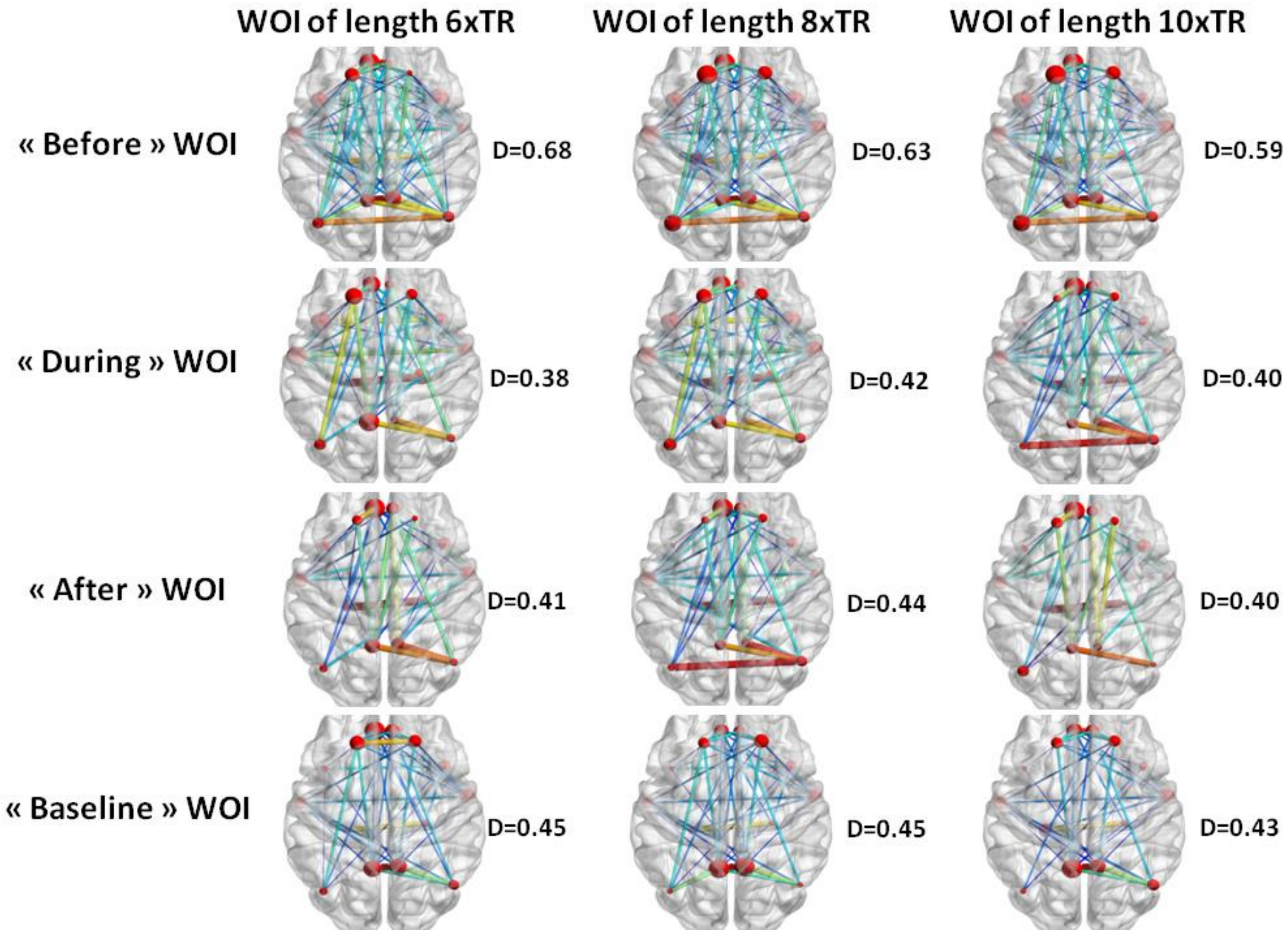

Supplementary figure: Maps of DMN intrinsic connectivity in the TLE group. The node sizes correspond to the number of connections for a node and the color lines show significant connections between pairs of regions (FDR-corrected $p < 0.05$). The variable D represents the network density (i.e. the number of significant connections divided by the total number of connections). Results from 3 window lengths (L = 6 TRs, 8 TRs and 10 TRs) were displayed and no significant difference of the network densities was observed between two lengths of windows (6 TRs vs 8 TRs; 6 TRs vs 10 TRs and 8 TRs vs 10 TRs).
